# Palliative Care in the Ageing European Population: A Cross-Country Comparison

**DOI:** 10.3390/ijerph21010113

**Published:** 2024-01-19

**Authors:** Giovanni Cerullo, Teodora Figueiredo, Constantino Coelho, Cláudia Silva Campos, António Videira-Silva, Joana Carrilho, Luís Midão, Elísio Costa

**Affiliations:** 1 Palliative Care, Centro Hospitalar Universitário do Algarve, 8000-386 Algarve, Portugal; gcerullo@chalgarve.min-saude.pt; 2 Faculty of Medicine, University of Coimbra, 3000-548 Coimbra, Portugal; 3 Faculty of Medicine, University of Porto, 4200-319 Porto, Portugal; 4 CINTESIS@RISE, Biochemistry Lab, Faculty of Pharmacy, University of Porto, 4050-313 Porto, Portugal; tgfigueiredo@ff.up.pt (T.F.); cccoelho@ff.up.pt (C.C.); cscampos@ff.up.pt (C.S.C.); jcarrilho@ff.up.pt (J.C.); lmidao@ff.up.pt (L.M.); 5 Department of Biological Sciences, Faculty of Pharmacy, University of Porto, 4050-313 Porto, Portugal; 6 Porto4Ageing—Competences Centre on Active and Healthy Ageing, Faculty of Pharmacy, University of Porto, 4050-313 Porto, Portugal; 7 Pediatric University Clinic, Faculty of Medicine, University of Lisbon, 1649-028 Lisbon, Portugal; antonioascenso@campus.ul.pt; 8 Research Centre in Physical Activity, Health and Leisure, Faculty of Sport, University of Porto, 4200-450 Porto, Portugal; 9 Centro de Investigação em Desporto, Educação Física, Exercício e Saúde (CIDEFES), Universidade Lusófona, 1749-024 Lisbon, Portugal

**Keywords:** palliative care, end of life, older adults, SHARE, healthcare disparities

## Abstract

With Europe’s ageing population and rising demand for palliative care, it is crucial to examine the use of palliative care among older adults during their last years of life and understand the factors influencing their access and end-of-life circumstances. This study employed a cohort of SHARE participants aged 65 years or older who had passed away between Wave 6 (2015) and Wave 7 (2017). Information on death circumstances, palliative care utilization, and associated variables were analysed. The study revealed that nearly 13.0% of individuals across these countries died under palliative care, with Slovenia having the lowest rate (0.3%) and France the highest (30.4%). Palliative care utilization in the last 30 days before death was observed in over 24.0% of participants, with the Czech Republic having the lowest rate (5.0%) and Greece the highest (48.8%). A higher risk of using or dying in palliative care was significantly associated with cognitive impairment (low verbal fluency), physical inactivity, and good to excellent self-perceived health. This work highlights the urgent need for enhanced global access to palliative care and advocates for the cross-country comparison of effective practices within Europe, tailored to the unique healthcare needs of older adults.

## 1. Introduction

In Europe, with the rising life expectancy and declining fertility rates, the percentage of older people is growing. In 2019, individuals aged 65 or over numbered 90.5 million, and projections indicate that this number will reach 129.6 million by 2050 [1]. Ageing is often accompanied by health conditions such as cardiovascular disease, osteoporosis, diabetes, dementia, and cognitive impairment, along with comorbidities, decreased mobility, and autonomy [2]. Older adults may also experience social changes due to the loss of roles both at home and in society. Reduced environmental stimulation results in social isolation and loneliness, contributing to mental health issues or a perceived decline in health [3].

This pronounced population ageing is imposing a significant economic burden and social challenges, including concerning palliative and end-of-life care. Alarmingly, the World Health Organization (WHO) estimates that only 12% of those in current need receive palliative care [4]. According to the WHO’s definition, palliative care is an approach to alleviate and prevent health-related suffering experienced by adults, children, and their families when confronted with life-threatening illnesses. This approach focuses on a holistic and individualized strategy, addressing not only the physical aspects but also the psychological, social, and spiritual dimensions of suffering [5].

Palliative medicine varies across European countries, with some countries having well-established and fully integrated palliative care services, including specialized teams and services available in hospitals, hospices, and dedicated palliative care units. Conversely, in other countries, palliative care is still in the developmental stages, and access to such services may be limited [5]. For instance, while the Netherlands, the United Kingdom, and Germany present a significantly advanced integration and coverage level of palliative care in different areas of their health systems, Romania, Serbia, Latvia, and Montenegro face substantial palliative needs. These countries have minimal to no palliative care activity, with limited integration capacity and specialized services in the field [6].

Even though effective implementation of palliative care within healthcare systems can be relatively cost-effective and lead to reduced healthcare costs of end-of-life care through reduced unnecessary hospitalizations and inappropriate diagnostics or interventions [7], its integration into European healthcare systems faces considerable impediments. A qualitative study across European countries revealed several barriers to integrating palliative care, including insufficient training, no official certification for professionals in the field, poor coordination and continuity of care for both patients and providers, omission from national regulatory frameworks, and disparities in palliative care laws and regulations among countries [8]. In fact, a recent report by the WHO highlights that only eight European countries have specific national laws incorporating palliative care [9].

Even where integrated programs exist, the timeliness of referrals from health professionals does not consistently align with maximizing benefits. This is attributed to factors such as not knowing that resources exist, ignorance regarding the nature of palliative care, reluctance to make referrals, stringent eligibility criteria for specialist palliative care services, and hesitancy on the part of patients and/or their families to accept referrals [10]. This reluctance observed among patients and families may vary due to cultural factors; however, a prevalent association between palliative care and the end-of-life phase is a common contributing factor [10].

Numerous studies have consistently demonstrated that referring patients to palliative care and implementing tailored palliative care interventions lead to significant enhancements in the quality of life for individuals facing advanced diseases [11,12,13,14]. Improving the quality of life assumes a central role in palliative care. In this context, quality of life is not about the absence of disease or suffering. Palliative care aims to assist patients in reevaluating their expectations and refocusing on critical aspects of life within the context of their illness trajectory [15]. Over the past decade, multiple definitions have emerged in attempts to delineate the concept of quality of life [16]. Furthermore, several sociodemographic, socioeconomic, psychosocial, and behavioural factors have been associated with the quality of life of older adults [17,18,19].

Despite advancements in enhancing the quality of life, research has shown that early specialized palliative care contributes to an overall improvement in patient satisfaction, empowering them with a great sense of control over their circumstances [20]. Palliative care has also been proven to substantially alleviate symptom burden and severity while concurrently enhancing the well-being of patients, even in the advanced stages of their diseases [14,21].

Furthermore, a systematic review has demonstrated that early palliative care for patients with advanced non-small-cell lung cancer can effectively mitigate depressive symptoms [22]. Additionally, the positive outcomes extend beyond the patients to include benefits for caregivers, family members, and friends. These individuals experience greater satisfaction with the quality of care provided and a heightened focus on addressing caregiver needs [23].

It is crucial to underscore that, despite the improvements in various aspects such as quality of life, patient and caregiver satisfaction, and the alleviation of symptoms and depressive feelings, the evidence regarding the association of palliative care with survival benefits is inconclusive [24,25]. However, findings from a recent cohort study involving patients with advanced lung cancer indicate a positive association between palliative care and survival, with early initiation of palliative care being specifically linked to increased survival rates [25].

Despite the established benefits of palliative care, it remains an underutilized resource, particularly among older adults, who exhibit distinct palliative care needs compared to the general population [26,27]. Consequently, there is a critical need to comprehend how and to what extent palliative care is utilized by the growing older population across different countries. This understanding is crucial for comparing and analyzing effective practices, as well as identifying barriers and facilitators to enhance access and outcomes in palliative care services tailored to the distinctive healthcare needs of this demographic. Addressing this gap is essential for developing targeted approaches that enhance the quality of end-of-life care for the older population, contributing to the advancement of palliative care strategies on a global scale.

In light of this, this study seeks to determine the prevalence of palliative care utilization and the occurrence of death within palliative care among older people in different European countries. Additionally, it aims to conduct a comparative analysis between those who received palliative care and those who did not, providing insights into the demographic profile and clinical characteristics of individuals who resort to palliative care services.

## 2. Materials and Methods

### 2.1. Study Participants

SHARE, short for Survey of Health, Ageing and Retirement in Europe, is a longitudinal cohort study encompassing community-dwelling individuals aged 65 years or older, spanning 27 European countries plus Israel. This study yields insights from a vast dataset comprising over 140,000 individuals. Since its commencement in 2004, SHARE has periodically disseminated data at two-year intervals, in 10 different waves. In this study, all individuals from Wave 6 (data from 2015) who had passed away by the data collection in Wave 7 (data from 2017) were included. In the Wave 7 interview, a proxy for the participant was interviewed, and data regarding the circumstances of their death were collected.

### 2.2. Palliative Care

One of the questions posed to the proxy of participants who had passed away was about the circumstances of their death, aiming to discern whether they had died in palliative care units. For proxies of participants who did not pass away in palliative care units, an additional inquiry was made regarding whether they had sought palliative care in the last 4 weeks of life. Based on this information, a variable with three categories was created: those who died in palliative care, those who sought palliative care in the last 4 weeks before death, and those who neither died in nor sought palliative care.

#### 2.2.1. Prevalence of Death in Palliative Care

All participants who passed away in a hospital in the palliative care unit, in an assisted living facility or a continuing care unit, or a palliative care unit were coded as having died in palliative care.

#### 2.2.2. Prevalence of Palliative Care Use in the 30 Days before Death

Participants who sought palliative care in the last 4 weeks of life were coded as having accessed palliative care in the last 4 weeks of life.

### 2.3. Associated Variables

The associated variables were grouped into five main categories:

1. Sociodemographic:
  a. “Gender” was a dichotomous variable with “Male” and “Female” as possible responses.
  b. “Age” was derived from the participant’s year of birth, referencing the year 2015. It was recoded into three age classes: “65–74 years”, “75–84 years”, and “85+”.
  c. “Marital Status”, initially with five possible responses, was recoded into “Divorced or Single”, “Married/Registered partnership”, and “Widowed”.
  d. “Years of Education” was a continuous variable with values ranging from 0 to 25 years.
  e. “Economic Situation” was subjectively assessed through the question, “How often do you think a lack of money prevents you from doing things you like to do?”, with possible responses being “Often”, “Sometimes”, “Rarely”, and “Never”.
2. Cognitive Performance was assessed as previously reported [28]:
  a. “Temporal Orientation” was assessed through four questions: “What day of the month is it?”, “What month is it?”, “What year is it?”, and “Can you tell me what day of the week it is?”. The score ranged from 0 to 4 and was determined by the number of correct answers. This result was dichotomized into two categories: a score of 3 or less, suggestive of impairment and signs of compromise, or a score of 4, representative of no impairment.
  b. “Numeracy” evaluates the participant’s mathematical performance, specifically through five items measuring subtraction skills, where participants were asked to subtract 7 from 100 and then continue subtracting from the given answer four more times. Numeracy scores ranged from 0 to 5 and were determined by the number of mathematical questions the participants answered correctly. In case of errors, subsequent answers were counted if they were correct about the previous number. This variable was dichotomized into two categories: a score of 3 or less, indicative of impairment, or a score greater than 3.
  c. The “Verbal Fluency” variable measured participants’ language ability and executive function through the question, “I would like you to name as many different animals as you can remember. You have one minute to do so”. Verbal fluency scores were based on the number of valid animal names the participant could recall. This variable was dichotomized into two categories: a score of more than 15 correct answers or a score of 15 or fewer, indicative of verbal fluency impairment.
  d. The participant’s “Memory” was assessed through verbal learning and recall tests. After hearing a list of ten words once, the participant was tested twice, once immediately (immediate recall) and once after 5 to 10 min (delayed recall). Total scores from the two tests ranged from 0 to 10 and were determined by the number of words the participant could remember each time. Scores of immediate recall <5 and scores of delayed recall <4 were indicative of impairment.
3. Physical Condition:
  a. “Limitations in Activities of Daily Living”: In SHARE, activities of daily living were assessed through an operationalized version of Katz, which includes six activities, with scores ranging from 0 to 6. The higher the score, the more difficulties with these activities, and the lower the mobility of the respondent. Responses were recoded into 3 groups: “0”, “1”, and “2 or more”.
  b. “Limitations in Instrumental Activities of Daily Living”: In SHARE, limitations in instrumental activities of daily living were assessed through an operationalized version of Lawton and Brody [29], which includes seven activities. Thus, the score ranges from 0 to 7. The higher the index, the more difficulties with these activities, and the lower the mobility of the respondent. Responses were recoded into 3 groups: “0”, “1”, and “2 or more”.
  c. “Body Mass Index” was calculated using the participant’s height and weight. The results were categorized into “BMI ≥ 30”, “25 ≤ BMI ≤ 29.9”, and “BMI ≤ 24.9”.
  d. “Physical inactivity was assessed through the questions, ‘We would like to know the type and amount of physical activity you do in your daily life. How often do you engage in physical activities that require a lot of physical effort, such as sports, heavy household chores, or a job that requires physical work?’ and ‘How often do you engage in activities that require a moderate level of energy, such as gardening, cleaning the car, or taking a walk?’ Responses were coded as ‘Engages in vigorous or moderate physical activity’ and ‘Does not engage in vigorous or moderate physical activity’.
4. Emotional State:
  a. “CASP”: This is a measure of quality of life in old age. CASP-12 is the 12-item version of CASP-19. The scale consists of four subscales, whose initials form the acronym: control (C), autonomy (A), self-realization (S), and pleasure (P). The 12 items are assessed on a four-point Likert scale (“often”, “sometimes”, “rarely”, “never”). The resulting score is the sum of these 12 items, ranging from 12 to 48, with higher scores indicating a higher quality of life.
  b. “EURO-D”: The EURO-D scale was originally developed to derive a common scale of depression symptoms from various depression instruments used in different European countries. The resulting scale consists of the following items: depression, pessimism, suicidal tendencies, guilt, sleep, interest, irritability, appetite, fatigue, concentration (in reading or entertainment), pleasure, and crying. The maximum score a participant can obtain is 12 (“very depressed”), and the minimum score is 0 (“not depressed”). The variable was dichotomized: a score on a scale of 4 or higher is categorized as “with depressive symptoms”, and a score on a scale below 4 is categorized as “without depressive symptoms”.
  c. “Loneliness”: The Three-Item Loneliness Scale [30] is a short version of the R-UCLA Loneliness Scale [31], which measures loneliness indirectly. The three items are answered on a three-point Likert scale (“often”, “sometimes”, “almost never or never”). The minimum score is 3 (“not lonely”), and the maximum is 9 (“very lonely”).
  d. “Life Satisfaction” was assessed through the question, “On a scale of 0 to 10, where 0 means totally dissatisfied and 10 means totally satisfied, tell us to what extent you are satisfied with your life?”. The response to this variable was dichotomized based on the median of the responses: people with values below the median were classified as “Dissatisfied”, and participants with values equal to or above the median were classified as “Satisfied”.
  e. “Satisfaction with Social Network” was assessed through the questions, “In general, on a scale of 0 to 10 where 0 means completely dissatisfied and 10 means completely satisfied, what is your satisfaction level with the relationships you have with the people we just talked about?” and “You mentioned that you don’t have anyone to talk to about important matters and that there’s no one important to you for any other reason. On a scale of 0 to 10, where 0 means extremely dissatisfied and 10 means extremely satisfied, what is your satisfaction level with this situation?”. The response to this variable was dichotomized based on the median of the responses: people with values below the median were classified as “Dissatisfied”, and participants with values equal to or above the median were classified as “Satisfied”.
5. Physical Health:
  a. “Self-perceived health” was assessed through the question, “Would you say your health is…” and was categorized as: “Poor or very poor”, “Good”, or “Very good or excellent”.
  b. “Chronic diseases” were based on the number of chronic diseases reported by each individual, which was dichotomized into “0 or 1 chronic disease” and “2 or more chronic diseases”.
  c. “Polypharmacy” was assessed through the question, “On a normal day, do you take at least five different medications? Please include medications prescribed by the doctor, over-the-counter medications, and dietary supplements such as vitamins and minerals”, with the question dichotomized into “Yes” and “No” [32].

### 2.4. Analysis

A descriptive analysis of the results was conducted to estimate the proportion of individuals who passed away between waves 6 and 7 and either died in palliative care or sought palliative care in their last 4 weeks of life. The prevalence standardized by age and gender of death in palliative care and the use of palliative care by country, along with 95% confidence intervals (95% CI), were also evaluated, with standardization of all results using the European standard population of 2013 (EUROSTAT, 2013).

With individuals grouped by country, a multilevel logistic regression was performed, with “death in palliative care” and “use of palliative care” as dependent variables. Initially, a univariate multilevel logistic regression model was run for each dependent variable, considering each covariate to identify potential associated factors (unadjusted model). Only significant covariates were included in a final multivariable multilevel logistic regression model (adjusted model). The country was treated as a random effect. Odds ratios (OR) and their 95% confidence intervals were reported. Missing data were imputed through multiple imputations, relying on regression models to predict missing values and incorporating uncertainty through an iterative approach. These analyses were all conducted using IBM SPSS (version 28), with a chosen significance level of *p* < 0.05.

### 2.5. Ethics Approval

The SHARE study underwent ongoing ethical scrutiny, and starting from wave 4, it received review and approval from the Ethics Council of the Max Planck Society. Subsequent to its execution with publicly accessible data, no further ethics approval was necessary for this study.

## 3. Results

From the 68,085 individuals surveyed in wave 6 of SHARE, our analysis encompasses the subset of participants who passed away by the time wave 7 was conducted. The present study included 2252 participants from 18 countries, comprising 47.1% women (n = 1061) and 52.9% men (n = 1191). Substantial heterogeneity was observed among the samples from each country, with the lowest number of participants in the Luxembourg sample (n = 25) and the highest number in the Spain sample (n = 333), representing 1.1% and 14.8% of the total sample, respectively. From the total sample, individuals who accessed palliative care in the last 30 days of life and those who died in palliative care were analysed.

### 3.1. Death in Palliative Care

**Figure 1:**
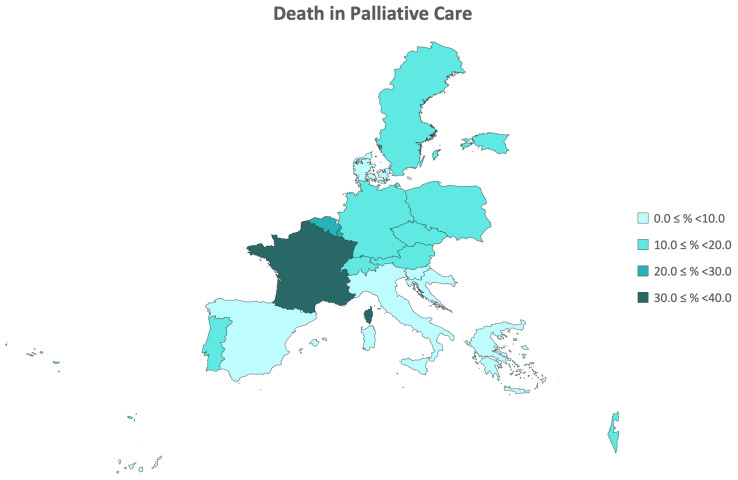
Regional distribution of palliative care death prevalence by country.

From the final sample of 2252 participants who passed away between Waves 6 and 7 of the SHARE study, nearly 13% of participants died under palliative care across the 18 countries included in Wave 6 of SHARE. This palliative care mortality rate ranged from 0.3% to 30.4%, with Denmark, Croatia, and Slovenia having the lowest rates, and Belgium, Luxembourg, and France having the highest rates. Overall, this palliative care mortality rate was higher in women than in men (13.8% vs. 11.1%). In the male gender, there was an observed trend of increasing rates with age, although this trend was not evident in the female gender. The prevalence of death in palliative care by country is detailed in Table 1 and demonstrated in the regional map of Europe in Figure 1.

### 3.2. Palliative Care Utilization up to 30 Days before Death

**Figure 2:**
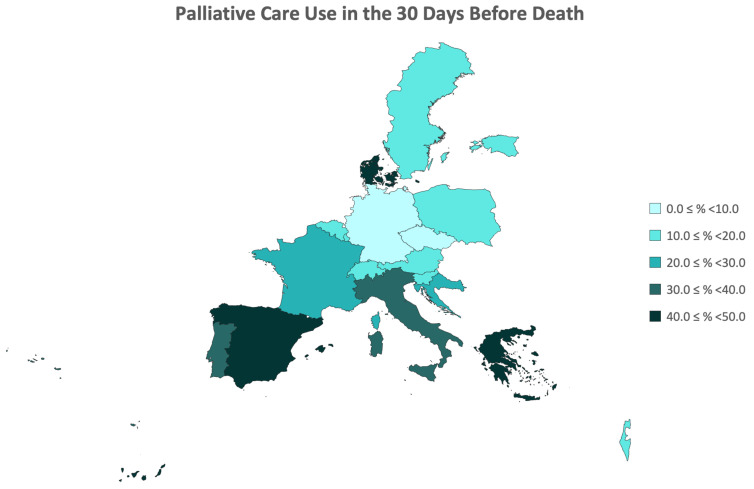
Regional Distribution of Prevalence of Palliative Care Utilization Up to 30 Days Before Death by Country.

Subsequently, an analysis of the prevalence of access to palliative care was conducted, with the data presented by country in Table 2 and illustrated in the regional map of Europe in Figure 2. From the final sample of 2252 participants who passed away between Waves 6 and 7 of the SHARE study, over 24% of participants sought palliative care in the 4 weeks preceding their death across the 18 countries included in Wave 6 of SHARE. This utilization rate of palliative care ranged from 5.0% to 48.8%, with the Czech Republic, Germany, and Slovenia having the lowest rates, and Denmark, Greece, and Spain having the highest rates. Specifically, there were no significant differences in the overall rates of accessing palliative care between the genders, indicating comparable percentages for women and men. Generally, there was an observed trend of increasing utilization rates with age, although this trend is not as linear in the male gender.

### 3.3. Associated Variables

#### 3.3.1. Death in Palliative Care

**Figure 3:**
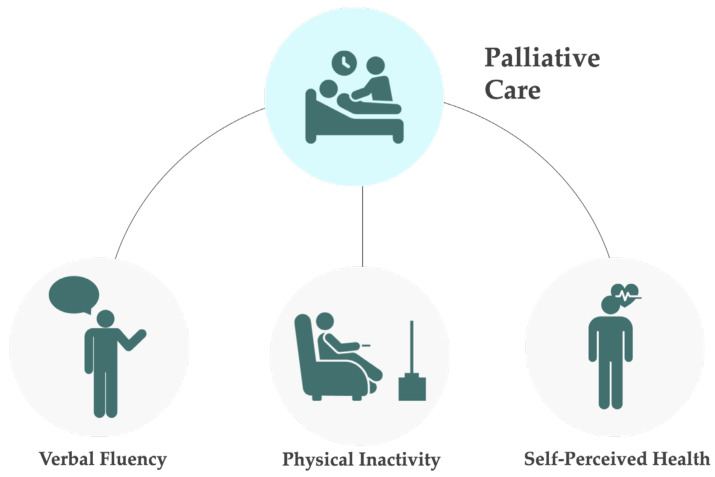
Schematic representation of the variables associated with a higher risk of dying in palliative care or using palliative care in the last 30 days before death. This risk was significantly associated with cognitive impairment (low verbal fluency), physical inactivity, and good to excellent self-perceived health.

From all the sociodemographic variables studied (gender, age, marital status, years of education, and economic situation), none showed a significant association with death in palliative care (Table 3). Among the cognitive performance variables (temporal orientation, numeracy, memory, and verbal fluency), only verbal fluency demonstrated an association, with individuals having a 1.457 times higher risk of dying in palliative care if they had cognitive impairment in this domain. Regarding physical condition variables (limitations performing ADLs and iADLs, BMI, and physical inactivity), only physical inactivity proved significant, with individuals who do not engage in moderate or vigorous physical activity having a 1.500 times higher risk of dying in palliative care compared to those who do. Among emotional state variables (quality of life and well-being, depression, loneliness, and life and network satisfaction), only depression was significant, with individuals exhibiting depressive symptoms having a 1.324 times greater risk of dying in palliative care compared to those without depressive symptoms. In the domain of physical health (self-perceived health, chronic diseases, and polypharmacy), only self-perceived health showed significance, with individuals who rated their health as “Very good or excellent” and “good” being at greater risk of dying in palliative care. In the multivariate model, only EURO-D lost significance, showing that the other variables and independently associated with death in palliative care. A schematic representation of the variables significantly associated with a higher risk of dying in palliative can be seen in Figure 3.

#### 3.3.2. Palliative Care Utilization up to 30 Days before Death

None of the sociodemographic variables included in the model (Table 3) showed a significant association with the utilization of palliative care in the last 4 weeks before the participants’ death. Among cognitive performance variables, only verbal fluency demonstrated an association, with individuals having a 1.357 times higher risk of resorting to palliative care if they had cognitive impairment in this variable. Regarding physical condition variables, only physical inactivity proved significant, with individuals who do not engage in moderate or vigorous physical activity having a 1.234 times higher risk of needing palliative care compared to those who do. Among emotional state variables, none showed significance. In the domain of physical health, none showed significance either. In the multivariate model, all variables retained significance. A schematic representation of the variables significantly associated with a higher risk of using palliative care in the last 30 days before death can be seen in Figure 3.

## 4. Discussion

Access to palliative care and the improvement of the quality of life during the later stages of life remains a pertinent and globally discussed topic [33,34]. This underscores the significance of this service in affording individuals a dignified end to their lives. Nevertheless, there is a notable disparity in the provision of this access across diverse countries, a notion substantiated by the findings of our study. The results suggest that various variables play a pivotal role in influencing the accessibility of the older population to palliative care and in influencing the conditions surrounding their end-of-life experiences.

We examined the prevalence of death in palliative care by identifying participants who passed away in specific settings, such as a hospital’s palliative care unit, an assisted living facility, a continuing care unit, or a designated palliative care unit. Additionally, we assessed the prevalence of palliative care use in the 30 days before death, identifying participants who sought palliative care during this period. Differential relationships between the palliative care death data and the access to palliative care within the last 30 days of life were then elucidated across country groups. Distinct patterns were identified, and country groups were categorized.

Nations where the percentage of individuals who died while under palliative care exceeds those who accessed it in the last 30 days of life were Austria, Germany, France, Belgium, Czech Republic, and Luxembourg. France was the country that exhibited the highest prevalence of death in palliative care, with consistently high results for the prevalence of access to palliative care in the last 30 days before death, suggesting that the country has high levels of access to palliative care. It was demonstrated that there was indeed a significant increase in access to palliative care between 2009 and 2013, consequently reducing the number of deaths in hospitals [35]. In fact, data substantiating the enduring nature of this expansion in subsequent years have been identified, demonstrating that France can meet a substantial demand for palliative care with a correspondingly high provision [36]. On the other hand, the Czech Republic demonstrated a diminished prevalence of access to palliative care within the final four weeks of life, coupled with higher numbers for the prevalence of death occurring in palliative care settings. This phenomenon may be ascribed to challenges associated with the accessibility of such services, notably marked by their insufficient integration into the national healthcare systems as of the year 2016 [37].

Curiously, Sweden and Estonia were the only countries where there was minimal variance between the percentage of deaths in palliative care and the percentage of access to palliative care in the last 30 days of life, suggesting good access to palliative treatments.

Moreover, countries that exhibited a higher number of individuals accessing palliative care in the last 30 days of life than the number of deaths in palliative care were Spain, Italy, Denmark, Greece, Switzerland, Israel, Poland, Portugal, Slovenia, and Croatia. Denmark and Greece, in particular, showed the most significant difference, with a lower percentage of deaths in palliative care (95% CI, ≤8%) compared to a higher percentage of access to palliative care in the last 4 weeks of life (95% CI, ≥40%). Both countries face high demands for palliative care but different approaches to respond to that demand. Denmark has reached an advanced stage of palliative care integration in its national health system, providing access to the general population. On the other hand, Greece (similar to Croatia) is still grappling with challenges to achieve a similar level of integration, currently offering isolated palliative care services, mostly funded by donors or direct payment [38,39]. This suggests that the data implying low mortality in palliative care and high prevalence of access to palliative in the last 30 days of life from these two countries may originate from different circumstances. Curiously, Sweden and Estonia were the only countries where there was minimal variance between the percentage of deaths in palliative care and the percentage of access to palliative care in the last 30 days of life, both handling percentages above 18%. Indeed, Sweden demonstrates a high standard in the assessment of palliative care accessibility. Conversely, Estonia exhibits limitations in this domain, being another case where elevated percentages in both the prevalence of death in palliative care and access to it within the last 30 days of life necessitate exploration of external factors [38].

In 2003, recognizing the importance of developing a coherent, fully integrated national policymaking framework for palliative care for all European citizens, the Council of Europe implemented Supplementary Recommendation 24 [40]. The ultimate intention was to encourage national governments to adopt concrete palliative care legislation measures or health policies. In light of this, most European countries reference palliative care in the national health law, but only eight countries have specific national laws or acts incorporating palliative care. These include France, Belgium, Luxembourg, Italy, Portugal, Albania, Germany, and Armenia [41]. Despite the presence of policies, differences in the adoption of palliative care among various countries can be linked to income disparities. In low and middle-income nations, access to palliative care is restricted due to various factors, such as the unavailability of treatments (e.g., medications), the costly setup of palliative care facilities in hospitals, the absence of proper clinical systems/guidelines for palliative care services, and insufficient prioritization of palliative care [42]. Moreover, these variations can also be associated with socio-cultural contexts. For instance, engaging in discussions about death and dying with patients may be deemed problematic or inappropriate [43].

According to Eurostat data, life expectancy at birth in Europe is higher for women (83.2 years) than for men (77.5 years) [44]. These findings align with the results obtained in this study, revealing higher prevalence rates of death in palliative care for men aged over 75 years compared to other age groups in men. This suggests that this demographic may be characterized by a higher burden of comorbidities, consequently necessitating an increased demand for palliative care services. This holds particularly true for countries with limited accessibility to palliative care, as their priority should be individuals with higher comorbidities, which are more characteristic in older people, exemplified by nations such as Croatia, the Czech Republic, Estonia, Greece, Poland, and Slovenia. On the other hand, countries with more advanced palliative care infrastructure do not appear to exhibit the same correlation, which could be explained by more universal access to this service available to the population, indicative of broader freedom of choice in seeking such services [36].

To conduct a comparative analysis between those who resort to or die in palliative care and those who do not among older adults, a comprehensive analysis was performed. This included the study of several sociodemographic variables, along with variables related to cognitive performance, physical condition, emotional state, and physical health. These variables were selected to provide a thorough understanding of the diverse aspects that may influence an individual’s choice/option regarding the utilization of palliative care and the circumstances surrounding their death.

According to a recent integrative review, there are significant disparities in access to palliative care between demographic groups, suggesting a complex and multifaceted landscape of access. These disparities are notably accentuated along racial and ethnic lines, with particular distinctions evident between underrepresented groups and non-Hispanic whites. The most predominant and consistently significant correlation across the reviewed studies revealed that the utilization of palliative care services tended to be linked with factors such as female gender, advanced age, and geographical proximity to an urban area with expansive health care systems [45].

It is pertinent to note that primary users of palliative care extend beyond individuals facing cancer diagnoses. Geriatric patients diagnosed with non-malignant conditions, such as dementia, circulatory system diseases, and/or chronic diseases, demonstrate a higher inclination toward palliative care utilization. Notably, patients with dementia constitute a substantial portion of this user group due to their heightened care dependency, particularly in the terminal phases of their life [46].

Surprisingly, in this study, none of the sociodemographic variables exhibited significant association with higher utilization of palliative care, despite a slight tendency of females and older individuals to use these services. However, a higher utilization of palliative care was associated with individuals having low verbal fluency and physical activity. These relations can also be understood through the lens of advanced illness. Individuals facing serious medical conditions often experience declines in cognitive function, such as low verbal fluency, and physical capacity [47,48]. Additionally, the symptom burden associated with serious illnesses, a common reason for seeking palliative care, may contribute to decreased physical activity and cognitive performance [49]. A habit of low physical activity can also lead to a confluence of factors that may necessitate palliative care utilization. Reduced physical activity can contribute to an overall decline in physical performance [50].

A higher risk of dying in palliative care was associated with low verbal fluency and physical activity, and good to excellent self-perceived health. Additionally, in the unadjusted model, there was also a correlation with depression, which aligns with established knowledge that individuals facing poor prognoses often experience depression [51]. Depression can not only affect patients but also impede healthcare providers in delivering optimal care, potentially leading to non-adherence to treatment plans and contributing to unfavourable outcomes in the context of palliative care [51]. Depression is often associated with physical symptoms such as changes in appetite, sleep disturbances, and fatigue. These physical manifestations can contribute to a decline in overall health [52]. Depression can also negatively affect immune function and lead to increased vulnerability to infections and other health complications, potentially accelerating the dying process [53].

As mentioned before, in the palliative care setting, cognitive impairment is prevalent and often attributed to advanced, life-threatening illnesses [47]. Therefore, the observed low verbal fluency may serve as an indicator of underlying challenges or declining health, explaining the increased risk of death in palliative care. Furthermore, individuals less physically active may experience poorer health, which may lead to adverse outcomes. Adhering to recommended levels of physical activity is associated with reduced health risks and improved survival [54].

Self-reported health should be seen as people’s perception of their health rather than a measure of true health [55]. Individuals who perceive their health as excellent may tend to postpone the utilization of medical services and not adhere to other health protective behaviours [56]. Consequently, these individuals may delay accessing healthcare until their health experiences a decline or until they reach advanced stages of a terminal illness. In this context, postponing the initiation of palliative care could be correlated with an elevated risk of mortality after entry into the palliative care program. In fact, it has already been shown that delaying entry into palliative care by at least one week is linked to a threefold increase in the odds of a patient dying in the hospital as opposed to being discharged alive [57].

This study possesses notable strengths, notably a large participant pool and representation from 18 countries, providing a comprehensive overview of conditions in Europe. The uniformity of the SHARE across countries, with direct translations, facilitates robust comparisons. It also has several limitations, mainly due to the use of self-reported surveys and proxy respondents’ data in the SHARE. Concerns about data reliability arise due to potential inaccuracies resulting from individuals’ memory recall, subjective perception, and reporting biases. Participation bias may also influence results, as healthier individuals might be more inclined to participate than older individuals with comorbidities. Additionally, the study’s scope is constrained by the predetermined set of questions in the SHARE database, limiting adaptability to specific research needs. It should be also noted that the data do not include the number of individuals who accessed palliative care more than 4 weeks before the end of life, nor do they consider the quality of health (such as long-term illnesses or poverty) preceding the last two years at the end of life. The utilization of cross-sectional data may restrict the establishment of causal relationships and temporal sequence understanding. In addition to these limitations, it is important to note that the study was limited to a sample of individuals from 17 European countries and Israel, and the findings may not be generalizable to other populations. Awareness of these limitations is crucial for a nuanced interpretation of the findings.

## 5. Conclusions

With this work, and through the use of a cohort of SHARE participants, it was possible to highlight the European disparities in access to palliative care among older adults. Noteworthy variations in palliative care provision were identified across countries. Variances in the relationship between deaths in palliative care and access to it in the last 30 days of life were observed, emphasizing diverse national approaches. Factors such as low verbal fluency, reduced physical activity, depression, and self-perceived excellent health were linked to increased palliative care utilization and/or mortality risks. Despite the study’s strengths, including a large participant pool, it is crucial to recognize limitations in self-reported data and participation bias. This work underscores the global need for improved palliative care access and advocates for the cross-country comparison of effective practices within Europe. By identifying variations in palliative care utilization among older adults, this research emphasizes the importance of enhancing the integration and coverage of palliative care services. Such efforts should be tailored to address the distinct healthcare needs of this demographic, ultimately fostering a more comprehensive and equitable approach to end-of-life care across European nations.

## Figures and Tables

**Table 1:** Prevalence (%) by country, age, and gender of individuals who passed away in palliative care in the last years of life.

	Prevalence of Death in Palliative Care (%)											
	Total				Men				Women			
	Prevalence (95% CI)	Standardized Prevalence (95% CI)			Prevalence (95% CI)	Standardized Prevalence (95% CI)			Prevalence (95% CI)	Standardized Prevalence (95% CI)		
		65–74	75–84	≥85		65–74	75–84	≥85		65–74	75–84	≥85
Austria	13.7 (13.3–14.0)	16.7 (16.1–17.2)	10.5 (10.0–11.1)	9.3 (8.5–10.2)	14.2 (13.7–14.7)	21.4 (20.6–22.3)	4.8 (4.2–5.3)	8.3 (7.2–9.5)	12.5 (12.0–13.0)	10.0 (9.4–10.6)	17.6 (16.6–18.7)	9.7 (8.5–11.0)
Belgium	22.8 (22.3–23.3)	23.3 (22.7–24.0)	20.8 (20.0–21.6)	25.8 (24.4–27.2)	22.7 (22.0–23.4)	26.3 (25.3–27.3)	16.7 (15.7–17.7)	23.1 (21.2–25.0)	21.4 (20.7–22.0)	18.2 (17.4–19.0)	24.1 (23.0–25.4)	27.5 (25.5–29.6)
Croatia	3.4 (3.2–3.6)	0.0 (0.0–0.0)	8.6 (8.1–9.1)	4.2 (3.6–4.8)	2.0 (1.8–2.2)	0.0 (0.0–0.0)	5.9 (5.3–6.5)	0.0 (0.0–0.2)	4.6 (4.3–4.9)	0.0 (0.0–0.0)	11.1 (10.3–12.0)	6.7 (5.7–7.8)
Czech Republic	13.4 (13.1–13.8)	9.2 (8.8–9.7)	21.1 (20.3–21.9)	11.1 (10.2–12.1)	9.6 (9.1–10.0)	4.7 (4.2–5.1)	16.7 (15.7–17.7)	11.8 (10.5–13.2)	19.7 (19.1–20.4)	18.2 (17.4–19.0)	25.7 (24.5–27.0)	10.7 (9.5–12.1)
Denmark	4.8 (4.6–5.0)	8.3 (7.9–8.7)	0.0 (0.0–0.0)	2.3 (1.9–2.8)	4.8 (4.5–5.1)	7.7 (7.2–8.2)	0.0 (0.0–0.1)	4.8 (3.9–5.7)	4.9 (4.6–5.2)	9.1 (8.5–9.7)	0.0 (0.0–0.1)	0.0 (0.0–0.2)
Estonia	18.2 (17.8–18.6)	11.5 (11.0–11.9)	25.3 (24.4–26.1)	27.9 (26.5–29.4)	19.0 (18.4–19.6)	8.9 (8.3–9.5)	31.9 (30.6–33.3)	27.6 (25.6–29.7)	20.0 (19.3–20.6)	18.8 (17.9–19.6)	18.8 (17.7–19.8)	28.2 (26.2–30.4)
France	30.4 (29.9–31.0)	35.3 (34.5–36.1)	25.0 (24.1–25.9)	24.1 (22.8–25.5)	31.6 (30.8–32.4)	41.7 (40.4–42.9)	19.2 (18.2–20.3)	21.7 (19.9–23.6)	25.2 (24.5–25.9)	20.0 (19.2–20.9)	33.3 (31.9–34.8)	25.7 (23.8–27.8)
Germany	14.3 (14.0–14.7)	17.6 (17.1–18.2)	11.4 (10.9–12.0)	8.0 (7.2–8.8)	7.2 (6.8–7.6)	7.7 (7.2–8.2)	9.1 (8.4–9.9)	0.0 (0.0–0.2)	34.9 (34.1–35.7)	50.0 (48.7–51.4)	15.4 (14.4–16.4)	22.2 (20.4–24.2)
Greece	7.4 (7.2–7.7)	5.6 (5.2–5.9)	12.3 (11.7–12.9)	2.6 (2.2–3.1)	8.5 (8.1–8.9)	6.7 (6.2–7.2)	12.5 (11.7–13.4)	5.9 (5.0–6.9)	4.0 (3.8–4.3)	0.0 (0.0–0.0)	12.1 (11.3–13.0)	0.0 (0.0–0.2)
Israel	15.1 (14.8–15.5)	22.2 (21.6–22.9)	4.2 (3.8–4.5)	14.0 (13.0–15.1)	5.2 (4.9–5.6)	0.0 (0.0–0.0)	8.3 (7.6–9.1)	19.2 (17.6–21.0)	19.0 (18.4–19.6)	33.3 (32.2–34.5)	0.0 (0.0–0.1)	8.3 (7.2–9.5)
Italy	6.3 (6.1–6.6)	6.3 (5.9–6.6)	6.0 (5.6–6.4)	7.4 (6.7–8.2)	8.7 (8.3–9.1)	8.7 (8.1–9.3)	7.5 (6.8–8.2)	11.8 (10.5–13.2)	1.9 (1.7–2.1)	0.0 (0.0–0.0)	3.7 (3.3–4.2)	5.4 (4.5–6.4)
Luxembourg	24.0 (23.5–24.5)	30.0 (29.3–30.8)	12.5 (11.9–13.1)	28.6 (27.1–30.1)	21.2 (20.5–21.8)	33.3 (32.2–34.5)	0.0 (0.0–0.1)	25.0 (23.1–27.0)	26.1 (25.4–26.8)	25.0 (24.1–26.0)	25.0 (23.8–26.2)	33.3 (31.1–35.7)
Poland	10.8 (10.5–11.1)	9.1 (8.7–9.5)	12.9 (12.3–13.5)	12.5 (11.5–13.5)	9.4 (9.0–9.9)	7.1 (6.6–7.7)	12.5 (11.7–13.4)	11.1 (9.8–12.5)	12.9 (12.4–13.4)	12.5 (11.8–13.2)	13.3 (12.5–14.3)	13.3 (11.9–14.8)
Portugal	11.4 (11.0–11.7)	15.8 (15.3–16.3)	6.5 (6.0–6.9)	5.6 (4.9–6.2)	14.7 (14.2–15.3)	16.7 (15.9–17.5)	11.8 (10.9–12.6)	14.3 (12.8–15.8)	7.7 (7.3–8.1)	14.3 (13.6–15.0)	0.0 (0.0–0.1)	0.0 (0.0–0.2)
Slovenia	0.3 (0.2–0.4)	0.0 (0.0–0.0)	0.0 (0.0–0.0)	2.3 (1.9–2.7)	0.7 (0.6–0.8)	0.0 (0.0–0.0)	0.0 (0.0–0.1)	5.6 (4.7–6.6)	0.0 (0.0–0.0)	0.0 (0.0–0.0)	0.0 (0.0–0.1)	0.0 (0.0–0.2)
Spain	8.3 (8.0–8.6)	8.2 (7.8–8.6)	6.9 (6.5–7.4)	12.3 (11.4–13.4)	7.3 (6.9–7.7)	8.6 (8.0–9.2)	4.5 (4.0–5.1)	9.2 (8.1–10.5)	8.9 (8.5–9.4)	7.1 (6.6–7.7)	9.4 (8.6–10.2)	15.4 (13.9–17.0)
Sweden	19.8 (19.3–20.2)	16.7 (16.1–17.2)	22.6 (21.8–23.4)	25.4 (24.0–26.9)	15.6 (15.0–16.2)	10.0 (9.4–10.6)	20.0 (18.9–21.1)	27.6 (25.6–29.7)	25.5 (24.8–26.3)	25.0 (24.1–26.0)	27.3 (26.0–28.6)	23.3 (21.5–25.3)
Switzerland	10.9 (10.6–11.2)	7.7 (7.3–8.1)	14.8 (14.2–15.5)	14.3 (13.3–15.4)	8.1 (7.7–8.5)	0.0 (0.0–0.0)	20.0 (18.9–21.1)	11.1 (9.8–12.5)	23.3 (22.6–24.0)	33.3 (32.2–34.5)	8.3 (7.6–9.1)	20.0 (18.3–21.8)
TOTAL	12.9 (12.5–13.2)	11.7 (11.2–12.1)	12.6 (12.0–13.2)	13.6 (12.6–14.7)	11.1 (10.7–11.6)	10.2 (9.6–10.8)	11.8 (11.0–12.6)	13.6 (12.2–15.1)	13.8 (13.2–14.3)	14.8 (14.1–15.6)	13.5 (12.6–14.4)	13.7 (12.3–15.2)

**Table 2:** Prevalence (%) by country, age, and gender of individuals who sought palliative care up to 30 days before death.

	Prevalence of Palliative Care Use in the 30 Days before Death (%)											
	Total				Men				Women			
	Prevalence (95% CI)	Standardized Prevalence (95% CI)			Prevalence (95% CI)	Standardized Prevalence (95% CI)			Prevalence (95% CI)	Standardized Prevalence (95% CI)		
		65–74	75–84	≥85		65–74	75–84	≥85		65–74	75–84	≥85
Austria	11.7 (11.4–12.1)	12.5 (12.0–13.0)	7.9 (7.4–8.4)	18.6 (17.4–19.8)	5.8 (5.5–6.2)	0.0 (0.0–0.0)	14.3 (13.4–15.2)	8.3 (7.2–9.5)	19.0 (18.4–19.7)	30.0 (29.0–31.1)	0.0 (0.0–0.1)	22.6 (20.8–24.5)
Belgium	16.3 (15.9–16.7)	10.0 (9.6–10.4)	24.5 (23.7–25.4)	21.2 (20.0–22.5)	20.3 (19.7–20.9)	15.8 (15.0–16.6)	25.0 (23.8–26.2)	26.9 (24.9–29.0)	10.3 (9.8–10.7)	0.0 (0.0–0.0)	24.1 (23.0–25.4)	17.5 (15.9–19.2)
Croatia	29.9 (29.4–30.5)	29.2 (28.4–29.9)	31.4 (30.5–32.4)	29.2 (27.7–30.7)	28.2 (27.5–29.0)	26.3 (25.3–27.3)	29.4 (28.1–30.8)	33.3 (31.1–35.7)	36.1 (35.2–36.9)	40.0 (38.8–41.2)	33.3 (31.9–34.8)	26.7 (24.7–28.8)
Czech Republic	5.0 (4.8–5.3)	4.6 (4.3–4.9)	4.2 (3.9–4.6)	8.9 (8.1–9.8)	5.4 (5.1–5.8)	7.0 (6.5–7.5)	2.8 (2.4–3.2)	5.9 (5.0–6.9)	3.3 (3.0–3.5)	0.0 (0.0–0.0)	5.7 (5.1–6.3)	10.7 (9.5–12.1)
Denmark	44.7 (44.1–45.4)	50.0 (49.0–51.0)	40.0 (38.9–41.1)	34.9 (33.3–36.6)	49.2 (48.2–50.2)	53.8 (52.5–55.3)	45.8 (44.2–47.5)	38.1 (35.7–40.6)	39.7 (38.8–40.6)	45.5 (44.2–46.8)	33.3 (31.9–34.8)	31.8 (29.6–34.1)
Estonia	18.1 (17.6–18.5)	19.7 (19.1–20.3)	17.9 (17.2–18.6)	11.8 (10.8–12.8)	14.0 (13.5–14.5)	17.8 (17.0–18.6)	10.6 (9.9–11.5)	6.9 (5.9–8.0)	23.8 (23.1–24.5)	25.0 (24.1–26.0)	25.0 (23.8–26.2)	15.4 (13.9–17.0)
France	25.3 (24.8–25.8)	17.6 (17.1–18.2)	34.1 (33.1–35.1)	34.5 (32.9–36.2)	27.4 (26.6–28.1)	16.7 (15.9–17.5)	38.5 (37.0–40.0)	43.5 (40.9–46.1)	23.7 (23.0–24.4)	20.0 (19.2–20.9)	27.8 (26.5–29.1)	28.6 (26.5–30.7)
Germany	8.8 (8.5–9.1)	11.8 (11.3–12.2)	5.7 (5.3–6.1)	4.0 (3.5–4.6)	12.1 (11.6–12.6)	15.4 (14.6–16.2)	9.1 (8.4–9.9)	6.3 (5.3–7.3)	0.0 (0.0–0.0)	0.0 (0.0–0.0)	0.0 (0.0–0.1)	0.0 (0.0–0.2)
Greece	48.8 (48.1–49.5)	61.1 (60.1–62.2)	32.9 (31.9–33.9)	38.2 (36.5–39.9)	46.8 (45.8–47.7)	60.0 (58.5–61.5)	27.5 (26.2–28.8)	41.2 (38.7–43.8)	53.6 (52.6–54.6)	66.7 (65.1–68.2)	39.4 (37.9–41.0)	35.7 (33.4–38.1)
Israel	19.8 (19.4–20.3)	11.1 (10.7–11.6)	29.2 (28.2–30.1)	32.0 (30.5–33.6)	29.2 (28.5–30.0)	33.3 (32.2–34.5)	25.0 (23.8–26.2)	23.1 (21.2–25.0)	16.5 (15.9–17.0)	0.0 (0.0–0.0)	33.3 (31.9–34.8)	41.7 (39.2–44.3)
Italy	32.9 (32.4–33.5)	34.4 (33.6–35.2)	28.4 (27.5–29.3)	38.9 (37.2–40.7)	33.1 (32.3–33.9)	34.8 (33.7–35.9)	25.0 (23.8–26.2)	47.1 (44.4–49.8)	33.6 (32.8–34.4)	33.3 (32.2–34.5)	33.3 (31.9–34.8)	35.1 (32.8–37.5)
Luxembourg	16.8 (16.4–17.2)	20.0 (19.4–20.6)	12.5 (11.9–13.1)	14.3 (13.3–15.4)	17.3 (16.7–17.9)	16.7 (15.9–17.5)	25.0 (23.8–26.2)	0.0 (0.0–0.2)	17.7 (17.1–18.3)	25.0 (24.1–26.0)	0.0 (0.0–0.1)	33.3 (31.1–35.7)
Poland	15.2 (14.8–15.6)	18.2 (17.6–18.8)	12.9 (12.3–13.5)	8.3 (7.6–9.2)	23.1 (22.4–23.7)	28.6 (27.6–29.6)	18.8 (17.7–19.8)	11.1 (9.8–12.5)	3.1 (2.8–3.3)	0.0 (0.0–0.0)	6.7 (6.1–7.3)	6.7 (5.7–7.8)
Portugal	31.7 (31.1–32.2)	31.6 (30.8–32.3)	35.5 (34.5–36.5)	22.2 (20.9–23.6)	19.3 (18.7–20.0)	25.0 (24.1–26.0)	17.6 (16.6–18.7)	0.0 (0.0–0.2)	46.8 (45.8–47.8)	42.9 (41.6–44.1)	57.1 (55.3–59.0)	36.4 (34.0–38.8)
Slovenia	10.2 (9.8–10.5)	10.7 (10.3–11.2)	9.7 (9.2–10.2)	9.1 (8.3–10.0)	11.7 (11.3–12.2)	15.8 (15.0–16.6)	5.4 (4.9–6.0)	11.1 (9.8–12.5)	6.3 (6.0–6.7)	0.0 (0.0–0.0)	16.0 (15.0–17.0)	7.7 (6.6–8.9)
Spain	41.7 (41.0–42.3)	44.9 (44.0–45.8)	39.2 (38.2–40.3)	34.4 (32.8–36.1)	43.3 (42.4–44.2)	45.7 (44.4–47.0)	40.9 (39.4–42.5)	39.5 (37.0–42.0)	39.4 (38.5–40.2)	42.9 (41.6–44.1)	37.5 (36.0–39.0)	29.5 (27.4–31.7)
Sweden	18.9 (18.5–19.3)	16.7 (16.1–17.2)	22.6 (21.8–23.4)	18.6 (17.5–19.9)	16.4 (15.8–16.9)	10.0 (9.4–10.6)	25.0 (23.8–26.2)	20.7 (18.9–22.6)	21.7 (21.0–22.3)	25.0 (24.1–26.0)	18.2 (17.2–19.2)	16.7 (15.1–18.3)
Switzerland	19.5 (19.1–20.0)	23.1 (22.4–23.7)	18.5 (17.8–19.3)	7.1 (6.4–7.9)	21.1 (20.4–21.7)	20.0 (19.2–20.9)	26.7 (25.4–28.0)	11.1 (9.8–12.5)	20.7 (20.1–21.4)	33.3 (32.2–34.5)	8.3 (7.6–9.1)	0.0 (0.0–0.2)
TOTAL	24.3 (23.8–24.8)	23.1 (22.5–23.8)	24.1 (23.3–25.0)	25.1 (23.7–26.5)	24.0 (23.3–24.7)	24.0 (23.1–25.0)	23.1 (22.0–24.3)	26.4 (24.5–28.5)	24.1 (23.4–24.8)	21.3 (20.4–22.2)	25.2 (24.0–26.5)	24.1 (22.2–26.1)

**Table 3:** Associated Variables for Death in Palliative Care and Use of Palliative Care in the 30 Days Before Death.

			Death in Palliative Care							Palliative Care Use in the 30 Days before Death						
		N Total	N (%) People Who Died in Palliative Care	Unadjusted Model			Adjusted Model			N (%) People Who Used Palliative Care up to 30 Days before Dying	Unadjusted Model			Adjusted Model		
		2252	288 (12.8)	OR	CI 95	*p*	OR	CI 95	*p*		OR	CI 95	*p*	OR	CI 95	*p*
Sociodemographic	Gender															
	Male	1191	142 (11.9)	1	-	-	-	-	-	291 (24.4)	1	-	-	-	-	-
	Female	1061	146 (13.8)	1.183	0.918–1.524	0.195	-	-	-	256 (24.1)	1.012	0.830–1.233	0.117	-	-	-
	Age															
	65–74 years	480	56 (11.7)	1	-	-	-	-	-	111 (23.1)	1	-	-	-	-	-
	75–84 years	900	113 (12.6)	1.108	0.782–1.571	0.564	-	-	-	217 (24.1)	1.074	0.822–1.402	0.603	-	-	-
	≥85 years	872	119 (13.6)	1.246	0.880–1.762	0.215	-	-	-	219 (25.1)	1.156	0.885–1.512	0.287	-	-	-
	Marital status															
	Single/Divorced	210	30 (14.3)	1	-	-	-	-	-	47 (22.4)	1	-	-	-	-	-
	Married/Registered partnership	1209	153 (12.7)	0.918	0.595–1.415	0.698	-	-	-	317 (26.2)	1.214	0.849–1.736	0.288	-	-	-
	Widowed	833	105 (12.6)	0.854	0.546–1.337	0.490	-	-	-	183 (22.0)	0.950	0.655–1.379	0.788	-	-	-
	Years of education															
	0–25	2252	288 (12.8)	1.002	0.973–1.032	0.879	-	-	-	547 (24.3)	0.979	0.956–1.002	0.068	-	-	-
	Economic situation															
	Often	491	60 (12.2)	1	-	-	-	-	-	124 (25.3)	1	-	-	-	-	-
	Sometimes	504	53 (10.5)	0.810	0.542–1.209	0.301	-	-	-	116 (23.0)	0.857	0.637–1.154	0.309	-	-	-
	Rarely	817	112 (13.7)	1.146	0.812–1.617	0.438	-	-	-	205 (25.1)	1.015	0.779–1.322	0.912	-	-	-
	Never	440	63 (14.3)	1.172	0.794–1.730	0.424	-	-	-	102 (23.2)	0.918	0.674–1.250	0.588	-	-	-
Cognitive performance	Temporal orientation															
	No impairment	1391	170 (12.2)	1	-	-	-	-	-	331 (23.8)	1	-	-	-	-	-
	Impairment	861	118 (13.7)	1.172	0.905–1.518	0.228	-	-	-	216 (25.1)	1.102	0.9001.350	0.347	-	-	-
	Numeracy															
	No impairment	1091	126 (11.5)	1	-	-	-	-	-	261 (23.9)	1	-	-	-	-	-
	Impairment	1161	162 (14.0)	1.269	0.984–1.639	0.067	-	-	-	286 (24.6)	1.082	0.888–1.318	0.434	-	-	-
	Memory															
	No impairment	337	42 (12.5)	1	-	-	-	-	-	75 (22.3)	1	-	-	-	-	-
	Impairment	1915	246 (12.8)	1.077	0.753–1.539	0.686	-	-	-	472 (24.6)	1.157	0.872–1.535	0.313	-	-	-
	Verbal fluency															
	No impairment	723	77 (10.7)	1	-	-	1	-	-	154 (21.3)	1	-	-	1	-	-
	Impairment	1529	211 (13.8)	1.457	1.098–1.934	0.009	1.276	1.119–1.454	<0.01	393 (25.7)	1.357	1.093–1.685	0.006	1.300	1.238–1.365	<0.001
Physical condition	Limitations performing ADLs															
	0	1187	142 (12.0)	1	-	-	-	-	-	284 (23.9)	1	-	-	-	-	-
	1	284	33 (11.7)	0.921	0.611–1.389	0.695	-	-	-	59 (20.8)	0.823	0.597–1.136	0.237	-	-	-
	2 or more	781	113 (14.5)	1.305	0.993–1.715	0.056	-	-	-	204 (26.1)	1.178	0.951–1.459	0.133	-	-	-
	Limitations performing iADLs															
	0	880	100 (11.4)	1	-	-	-	-	-	216 (24.5)	1	-	-	-	-	-
	1	195	23 (11.8)	1.109	0.676–1.819	0.683	-	-	-	55 (28.2)	1.227	0.859–1.754	0.260	-	-	-
	2 or more	1177	165 (14.0)	1.264	0.964–1.659	0.091	-	-	-	276 (23.4)	0.979	0.794–1.207	0.844	-	-	-
	Body mass index															
	IMC ≥ 30.0	424	50 (11.8)	1	-	-	-	-	-	95 (22.4)	1	-	-	-	-	-
	25 ≤ IMC ≤ 29.9	793	98 (12.4)	1.060	0.732–1.535	0.759	-	-	-	179 (22.6)	1.019	0.764–1.359	0.068	-	-	-
	IMC ≤ 24.9	1035	140 (13.5)	1.256	0.883–1.787	0.205	-	-	-	273 (26.4)	1.289	0.981–1.694	0.899	-	-	-
	Physical inactivity															
	Other	1139	124 (10.9)	1	-	-	1	-	-	262 (23.0)	1	-	-	1	-	-
	Never vigorous nor moderate physical activity	1113	164 (14.7)	1.500	1.161–1937	0.002	1.310	1.160–1.479	<0.01	285 (25.6)	1.234	1.012–1.503	0.037	1.151	1.101–1.204	<0.001
Emotional status	CASP															
	CASP-12	2252	288 (12.8)	0.968	0.970–1.003	0.108	-	-	-	547 (24.3)	0.990	0.977–1.003	0.140	-	-	-
	EURO-D															
	Without depressive symptoms	989	112 (11.3)	1	-	-	-	-	-	229 (23.2)	1	-	-	-	-	-
	With depressive symptoms	1263	176 (13.9)	1.324	1.022–1.716	0.034	-	-	-	318 (25.2)	1.170	0.958–1.429	0.123	-	-	-
	Loneliness															
	R-UCLA	2252	288 (12.8)	0.986	0.905–1.073	0.739	-	-	-	547 (24.3)	0.952	0.889–1.018	0.151	-	-	-
	Life satisfaction															
	Satisfied	1341	164 (12.2)	1	-	-	-	-	-	329 (24.5)	1	-	-	-	-	-
	Unsatisfied	911	124 (13.6)	1.127	0.872–1.456	0.361	-	-	-	218 (23.9)	0.988	0.807–1.208	0.903	-	-	-
	Network satisfaction															
	Satisfied	1396	180 12.9)	1	-	-	-	-	-	335 (24.0)	1	-	-	-	-	-
	Unsatisfied	856	108 (12.6)	0.986	0.759–1.281	0.917	-	-	-	212 (24.8)	1.040	0.849–1.274	0.703	-	-	-
Physical health	Self-perceived health															
	Poor or fair	118	5 (4.2)	1	-	-	1	-	-	33 (28.0)	1	-	-	-	-	-
	Good	374	41 (11.0)	2.722	1.039–7.129	0.042	2.612	1.705–4.001	<0.01	92 (24.6)	0.925	0.578–1483	0.747	-	-	-
	Very good or excellent	1760	242 (13.8)	3.533	1.415–8.818	0.007	2.949	1.960–4.439	<0.01	422 (24.0)	0.933	0.613–1.422	0.748	-	-	-
	Chronic Diseases															
	2 or more	1653	213 (12.9)	1	-	-	-	-	-	397 (24.0)	1	-	-	-	-	-
	0 or 1	599	75 (12.5)	0.982	0.736–1.311	0.902	-	-	-	150 (25.0)	1.054	0.844–1.316	0.644	-	-	-
	Polypharmacy															
	Yes	1171	144 (12.3)	1	-	-	-	-	-	287 (24.5)	1	-	-	-	-	-
	No	1081	144 (13.3)	1.093	0.848–1.409	0.491	-	-	-	260 (24.0)	0.990	0.813–1.207	0.922	-	-	-

## Data Availability

The data presented in this study are openly available at http://www.share-project.org/ (accessed on 18 March 2023) (SHARE Wave 6—DOI: 10.6103/SHARE.w6.710; SHARE Wave 7—DOI: 10.6103/SHARE.w7.711).
